# Equity in access to safely managed sanitation and prevalence of diarrheal diseases in Bangladesh: a national and sub-national analysis

**DOI:** 10.1186/s12879-022-07884-4

**Published:** 2022-11-21

**Authors:** Jahanara Akter, Md. Rashedul Islam, Shamima Akter, Md. Mizanur Rahman, Fahima Hossain, Md Rifat Anam, Md. Ashraful Alam, Papia Sultana, Shahedur Rashid

**Affiliations:** 1 Global Public Health Research Foundation, Dhaka, Bangladesh; 2 Jahangirnagar University, Dhaka, Bangladesh; 3 Sreepur Muktizodha Rahmat Ali Government College, Gazipur, Bangladesh; 4 Hitotsubashi Institute for Advanced Study, Hitotsubashi University, Kunitachi, Japan; 5 Department of Global Health Policy, Graduate School of Medicine, The University of Tokyo, Tokyo, Japan; 6 University of Rajshahi, Rajshahi, Bangladesh

**Keywords:** Sanitation, Diarrheal disease, Inequality, Multi-level analysis

## Abstract

**Background:**

In Bangladesh, safely managed sanitation (SMS) coverage is low, and diarrheal disease is a significant health problem. This study estimated the inequality in access to SMS facilities at the national and sub-national levels and assessed the prevalence of diarrheal diseases in connection with these improved facilities.

**Methods:**

Data were extracted from the Bangladesh Demographic and Health Survey, conducted during 2017–2018. SMS was defined as using an improved sanitation facility, which designed to hygienically separate excreta from human contact and include the use of a flush toilet connected to piped sewer system, septic tank, ventilated improved pit latrine, pit latrine with a slab, and composting toilet. The slope index of inequality (SII) and multi-level regression models were used for inequality and risk factors of SMS respectively.

**Results:**

The national coverage of SMS was 44.0% (45.3% and 43.5% in urban and rural areas, respectively). At the sub-national level, the lowest and highest coverage of SMS was observed in Mymensingh (32.9%) and Chittagong (54.1%) divisions, respectively. The national level SII indicated that wealthy households had access to higher SMS by 60.8 percentage points than poor households. Additionally, greater inequality was observed in rural areas, which was 71.9 percentage points higher in the richest households than in the poorest households. The coverage gap between the rich and poor was highest in the Sylhet division (85.3 percentage points higher in rich than in poor) and lowest in Dhaka (34.9 percentage points). Old and highly educated household heads and richest households had better access to higher levels of adequate sanitation. After adjusting for confounding variables, the prevalence of diarrheal disease was 14.0% lower in the SMS user group than in their counterparts.

**Conclusion:**

Substantial inequalities in access to SMS exist at both national and sub-national levels of Bangladesh, with the prevalence of diarrhea being lower among SMS users. These findings may help to prioritize resources for reducing inequality and expanding the coverage of improved sanitation in Bangladesh.

## Introduction

Universal and equitable access to safely managed sanitation (SMS) is vital for ensuring health and well-being of all. Although in 2020, 54.0% of the world population (4.2 billion people) used a SMS service, more than 1.7 billion people still lacked basic sanitation services, i.e., private toilets or latrines [1]. Scarcity of improved sanitation poses both health and economic threats as it increases the risk of various diseases such as diarrhea, cholera, typhoid, schistosomiasis, and some cancers through exposure to carcinogens [2]. Annually, inadequate water, sanitation, and hygiene together cause around 60.0% of the total diarrheal deaths in low- and middle-income countries (LMICs), among which 432,000 are attributed to poor sanitation only [1]. In addition, near about 480,000 children die globally each year due to diarrhea from waterborne diseases [3], all of which emphasize how important SMS services are for healthy living and socioeconomic development.

Even though some parts of the world have made remarkable progress in increasing access to basic sanitation services, serious inequalities remain in several regions [4]. For instance, coverage of SMS is quite low in African and South Asian countries [4], particularly in Bangladesh [5], though it is one of the top performing countries in the Millenium Development Goal (MDG) era. According to the World Bank reports, Bangladesh successfully reduced open defecation from 34.0% in 1990 to just 1.0% of the national population in 2015, but the quality of sanitation and its link with human development remain a challenge [6, 7]. The rapid urbanization and population growth of the country could be an associated factor for the proliferation of informal settlements and thereby this environmental degradation.

Several studies have been conducted in Bangladesh on sanitation [8–11] and its benefits in rural Bangladesh [12–14]; however, these studies are limited to only describing the household trends in access to improved sanitation facilities. Furthermore, little research is available on the inequality in access to improved sanitation facilities at the national and sub-national levels in Bangladesh using demographic and socioeconomic measures. A deeper understanding of this issue is critical for the Bangladeshi policymakers to optimize resource allocation and disease control as well as prioritize the programs that are focused on preventing epidemics. Also, if these inequalities are not addressed adequately and promptly, they could pose a threat to the attainment of Sustainable Development Goal (SDG) 6.2 for sanitation by 2030. Therefore, to the best of our knowledge, this is the first study to assess the equity in access to SMS at national and sub-national levels in Bangladesh using the available data from Bangladesh Demographic and Health Survey (BDHS) 2017–2018 [15]. This study aimed to: (1) estimate the inequalities in access to SMS facilities at the national and sub-national levels of Bangladesh, (2) determine the association of demographic and socioeconomic indicators with inequalities in access to Bangladeshi sanitation systems, and (3) assess the prevalence of diarrheal diseases in relation to SMS facilities in Bangladesh.

### Study population

Bangladesh, with a population of nearly 149.8 million in 2011, is one of the most densely populated countries in the world (1015 people per sq km) [16]. The country has eight administrative divisions: south (Barishal), southeast (Chittagong), central (Dhaka), west (Khulna), mid-western corner (Rajshahi), central (Mymensingh), northwest (Rangpur), and east (Sylhet) [17]. These regions exhibit different geographical, demographic, environmental, gastronomic, and economic characteristics [18]. Other areas lag behind the socio-economic status of the southern part of Bangladesh [16, 19, 20]. Poverty, hunger, and other socio-economic indicators disproportionately impact people in the northwestern part of Bangladesh [16, 19, 20].

### Data and methodology

**Fig. 1:**
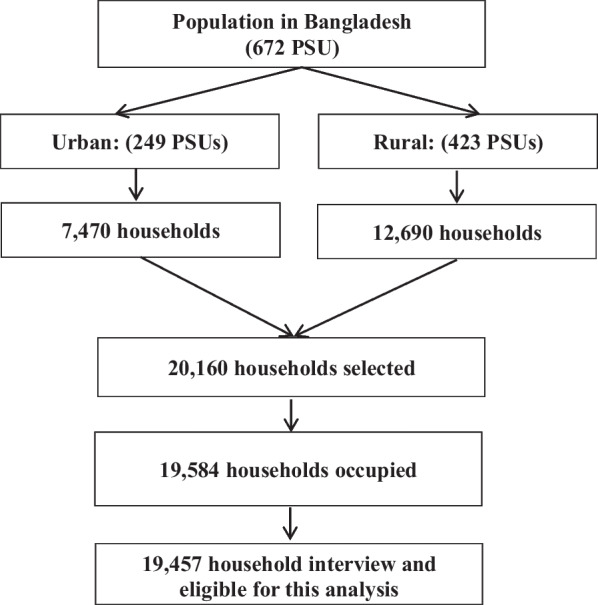
Schematic presentation of sample size calculation

Data for this study were extracted from the BDHS 2017–2018, a nationally representative cross-sectional study. This survey was conducted by the National Institute of Population Research and Training of the Ministry of Health and Family Welfare conducted between October 2017 and March 2018. It was based on a two-stage stratified sampling of households. In the first stage of sampling, a fixed number of primary sampling units (PSUs) were selected, with probability proportional to their size. In the second stage, households were selected within each primary sampling unit using equal probability systematic sampling. The BDHS is a representative probability sample of men and women based on a two-stage cluster sampling of households, stratified by rural and urban areas and eight administrative regions of the country. In the first stage of sampling, 675 PSUs were selected with a probability proportional to the PSU’s size. On an average, each PSU contained 120 households. In the second stage of sampling, 20,250 households were selected using a systematic random sampling method. Interviews were successfully completed in 19,584 households, of which 19,457 were eligible. The overall response rate was 99.4%. Figure 1 illustrates sample selection framework. The detailed research protocol, methods, and structured questionnaires are available on the DHS website [20].

### Outcomes

The outcome variables of this study were SMS and diarrheal diseases. According to the sanitation ladders of the WHO/UNICEF Joint Monitoring Program (JMP) [21, 22], SMS was defined as use of improved facilities that are not shared with other households and where excreta are safely disposed of in situ or removed and treated offsite. Moreover, an improved sanitation facility is designed to hygienically separate excreta from human contact and include the use of a flush toilet connected to piped sewer system, septic tank, ventilated improved pit latrine, pit latrine with a slab, and composting toilet. Conversely, an unimproved sanitation facility was defined as use of pit latrines without a slab or platform, hanging latrines or bucket latrines. According to the available BDHS data, improved sanitation facilities include flush or pour-flush to the piped sewer system, septic tank, or pit latrine, ventilated improved pit latrines, composting toilets, or pit latrines with slabs. Diarrhea was defined as the passage of three or more loose or liquid stools per day (or more frequent passage than is normal for the individual) [23] and the proportion of children under the age of five who had diarrhea in the 2 weeks preceding the survey were included.

### Covariates

We used individual, household, and community-level characteristics to assess the prevalence of diarrheal diseases. The variables were the household head’s age (≤ 24 years, 25–34 years, and ≥ 35 years), sex (male or female), and education (no education, primary, secondary education, and higher education), household’s wealth quintile (poorest, poorer, middle, rich, and richest), area of residence (urban or rural), and region of residence (Barisal, Chittagong, Dhaka, Khulna, Mymensingh, Rajshahi, Rangpur, and Sylhet).

### Statistical analysis

Descriptive statistics and frequency distributions were used to describe the participants’ characteristics. Two indices were used to summarize wealth-based inequalities in SMS coverage and the prevalence of diarrheal diseases: the slope index of inequality (SII) and the relative index of inequality (RII). Both indices were calculated using regression models that considered the whole population’s distribution of wealth. The SII expresses the absolute difference in coverage in percentage points and provides an idea of the actual efforts that will be needed to close the gap. By contrast, the RII measures the ratio of SMS coverage for poor and wealthy households at the national and sub-national levels and provides an idea about the degree of inequality. A multi-level logistic regression model was used to estimate the risk factors of SMS facilities, and Poisson regression was used to assess the prevalence of diarrheal diseases in connection sanitation variables. We used multi-level analysis because individuals were clustered within the same households, and households were clustered within communities in the BDHS data. The Stata MP (version 16.1) was used for all the statistical analyses. The level of statistical significance was set at *p* < 0.05. All analyses were adjusted for the probability weight. The probability weight is the invers probability of selection of each household into the study.

### Ethical considerations

The BDHS 2017–2018 was conducted by National Institute of Population Research and Training (NIPORT) and approved by the Institutional Review Boards (IRBs) at the ICF and the Bangladesh Medical Research Council (BMRC).

## Results

### Background characteristics

The background characteristics of the study population are presented in Table 1. In a total sample of 19,457 households, most individuals (77.5%) were ≥ 35 years old, and the majority of the household heads were male (84.6%). Of the total respondents, 40.7% fell into the poor category and most of them lived in rural areas (63.5%). Moreover, 27.8% had no formal education, and the Dhaka region accommodated the highest number of respondents (15.0%).

**Table 1:** Background characteristics of the study population, Bangladesh, 2017–2018

Characteristics	Frequency (N)	Percentage (%)
Age of household head (in years)		
≤ 24	616	3.2
25–34	3762	19.3
≥ 35	15,079	77.5
Gender of household head		
Male	16,464	84.6
Female	2993	15.4
Household education		
No education	5417	27.8
Primary	6315	32.5
Secondary	5008	25.7
Higher	2701	13.9
Household wealth quintile		
Poorest (Q1)	4077	21.0
Poorer (Q2)	3839	19.7
Middle (Q3)	3641	18.7
Rich (Q4)	3798	19.5
Richest (Q5)	4102	21.1
Area of residence		
Urban	7103	36.5
Rural	12,354	63.5
Region of residence		
Barisal	2053	10.6
Chittagong	2644	13.6
Dhaka	2912	15.0
Khulna	2517	12.9
Mymensingh	2217	11.4
Rajshahi	2563	13.2
Rangpur	2467	12.7
Sylhet	2084	10.7
Total	19,457	100.0

### Access to safely managed sanitation

**Fig. 2:**
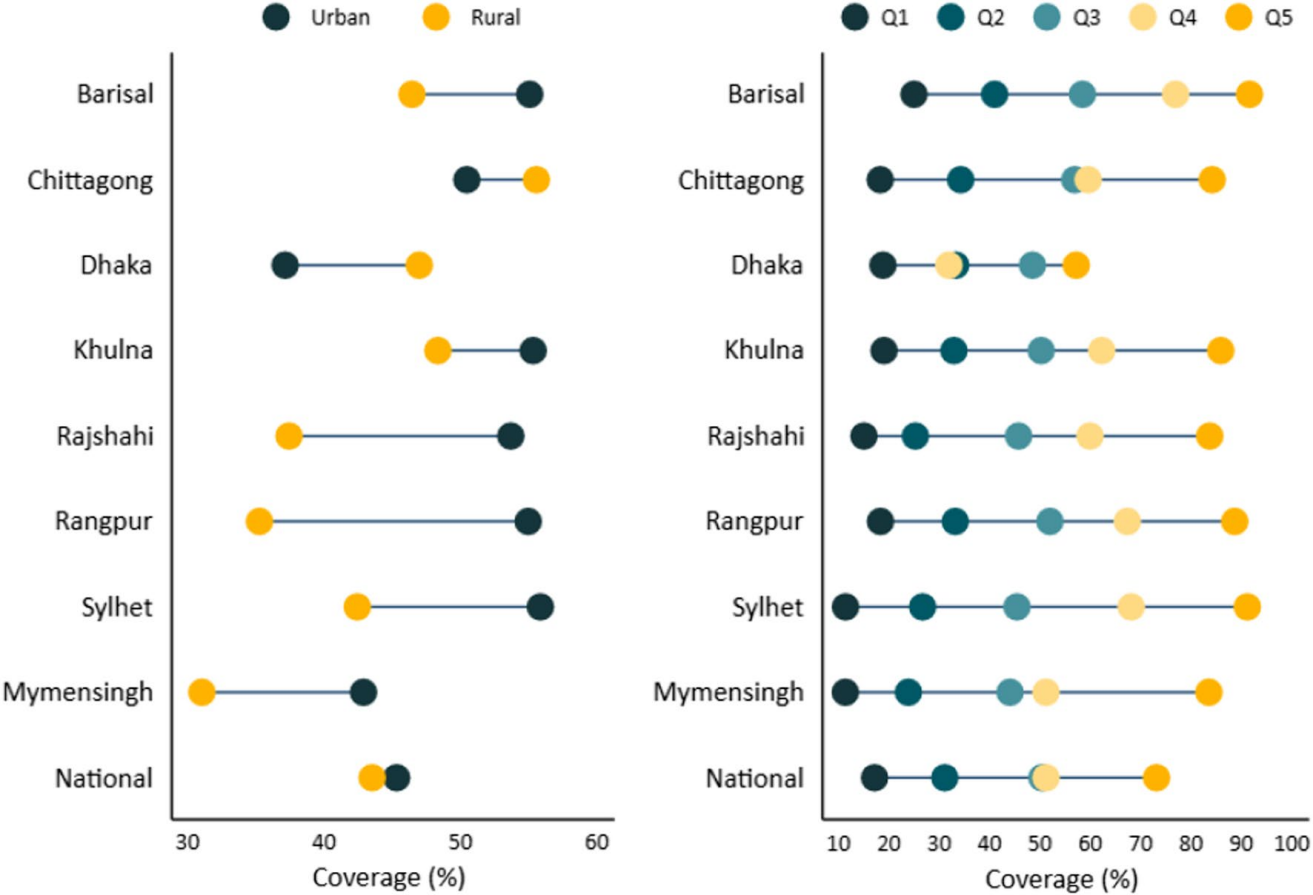
Residence-specific and wealth quintile-specific coverage of safely managed sanitation at national and sub national level, Bangladesh

Table 2 presents the national and sub-national coverage of sanitation facilities in Bangladesh. The national coverage of SMS was 44.0% (95% CI 43.3–44.7), whereas it was 45.3% (95% CI 44.0–46.6) and 43.5% (95% CI 42.6–44.3) in urban and rural areas, respectively. Among the eight administrative divisions, the lowest coverage was observed in Mymensingh (32.9%) and the highest in the Chittagong division (54.1%). Access to basic sanitation facilities varied substantially across all divisions in Bangladesh, with a sanitation coverage rate of 47.8% (95% CI 44.9–50.8) in Barisal, 49.8% (95% CI 47.8–51.9) in Khulna, 40.7% (95% CI 38.8–42.5) in Rajshahi, 38.0% (95% CI 36.1–40.0) in Rangpur, and 44.8% (95% CI 41.9–47.7) in Sylhet. Figure 2 presents the residence-specific and quintile-specific coverage of safely managed sanitation at the national and sub-national levels in Bangladesh.

**Table 2:** Inequality in access to safely managed sanitation at national and sub-national levels, Bangladesh

Region	Overall percent (95% CI)	Percent (95% CI)		Inequality indices (95% CI)	
		Poorest (Q1)	Richest (Q5)	SII	RII
National	44.0 (43.3–44.7)	17.0 (15.9–18.2)	73.0 (71.6–74.4)	60.8 (56.3–65.3)	5.0 (4.3–5.7)
Area of residence					
Urban	45.3 (44.0–46.6)	21.7 (17.9–26.1)	64.5 (62.7–66.4)	55.6 (47.9–63.2)	4.0 (3.1–5.0)
Rural	43.5 (42.6–44.3)	16.5 (15.4–17.8)	89.8 (88.0–91.4)	71.9 (69.2–74.5)	8.0 (6.9–9.1)
Region of residence					
Barisal	47.8 (44.9–50.8)	24.9 (20.7–29.6)	91.5 (83.4–95.8)	68.3 (62.0–74.5)	5.7 (4.2–7.2)
Chittagong	54.1 (52.4–55.8)	18.2 (15.0–21.8)	84.1 (81.4–86.4)	68.9 (61.7–76.0)	4.8 (3.6–6.0)
Dhaka	41.8 (40.5–43.2)	18.7 (15.4–22.4)	57.1 (54.7–59.4)	34.9 (23.5–46.2)	2.4 (1.7–3.1)
Khulna	49.8 (47.8–51.9)	18.9 (15.0–23.5)	85.8 (81.8–89.0)	67.2 (61.0–73.4)	5.1 (3.9–6.4)
Mymensingh	32.9 (30.6–35.2)	11.2 (8.8–14.3)	83.4 (76.5–88.6)	66.7 (58.0–75.4)	11.9 (6.8–17.0)
Rajshahi	40.7 (38.8–42.5)	14.9 (12.3–17.9)	83.6 (79.1–87.3)	69.2 (63.8–74.6)	8.2 (5.7–10.7)
Rangpur	38.0 (36.1–40.0)	18.2 (15.9–20.8)	88.5 (83.0–92.4)	67.6 (61.8–73.3)	8.7 (5.5–11.8)
Sylhet	44.8 (41.9–47.7)	11.3 (8.1–15.4)	91.0 (86.3–94.2)	85.3 (81.0–89.5)	17.0 (10.1–24.0)

*SII* slope index of inequality, *RII* relative index of inequality, *CI* confidence interval

Greater inequalities in access to SMS facilities were observed at the national and sub-national levels (Table 2). The national level SII indicated that access to SMS in wealthy households was 60.8 percentage points higher than that of disadvantaged households. Additionally, greater wealth inequalities were observed at the sub-national level. At the sub-national level, inequality was higher in the Sylhet division and lower in the Dhaka division, which was 85.3 and 34.9 percentage points, respectively, greater in the richest household than in the disadvantaged households. Furthermore, greater inequality was observed in rural areas, which was 71.9 percentage points higher in the richest households than in the poorest households.

### Risk factors associated with access to improved sanitation facilities

Table 3 presents the results of the multi-level logistic regression models for SMS. Higher age of the household heads was associated with higher odds of having access to improved sanitation facilities, by nearly 2.5-times in the age group ≥ 35 years (OR 2.36, 95% CI 1.82–3.07), followed by 25–34 years (OR 1.45, 95% CI 1.10–1.92). Gender of the household heads was associated with SMS, with female household heads having higher odds (OR 1.15, 95% CI 1.02–1.29) compared to male household heads. Similarly, household education was also an associated factor for improved sanitation facilities, with greater by nearly 2.5 times was observed with household heads having a higher education (OR 2.18, 95% CI 1.81–2.62), secondary education (OR 1.27, 95% CI 1.13–1.43), and primary education (OR 1.17, 95% CI 1.06–1.30) compared to those without education. The household wealth quintile displayed a significant association between having access to SMS, with higher odds in the richest quintile, which was more than 50-fold compared to the poorest quintile. The rural households had 2.55 times (95% CI 2.11–3.08) higher odds of having access to SMS compared to the urban households. Households from Mymensingh (OR 1.58, 95% CI 1.15–2.17), Sylhet (OR 1.91, 95% CI 1.35–2.70), Rajshahi (OR 2.06, 95% CI 1.48–2.87), Chittagong (OR 2.44, 95% CI 1.72–3.45), Khulna (OR 2.47, 95% CI 1.79–3.41), and Rangpur (OR 2.71, 95% CI 1.96–3.76) had higher odds of having access to SMS than those from Dhaka.

**Table 3:** Risk factors of safely managed sanitation: a multi-level analysis

Characteristics	Odds ratio	95% CI	*p*
Age of household head (in years)			
≤ 24	1.00		
25–34	1.45	1.10–1.92	< 0.01
≥ 35	2.36	1.82–3.07	< 0.001
Gender of household head			
Male	1.00		
Female	1.15	1.02–1.29	< 0.05
Household education			
No education	1.00		
Primary	1.17	1.06–1.30	< 0.01
Secondary	1.27	1.13–1.43	< 0.001
Higher	2.18	1.81–2.62	< 0.001
Household quintile			
Poorest	1.00		
Poorer	2.53	2.21–2.88	< 0.001
Middle	6.08	5.22–7.08	< 0.001
Rich	9.89	8.30–11.78	< 0.001
Richest	50.52	40.63–62.80	< 0.001
Area of residence			
Urban	1.00		
Rural	2.55	2.11–3.08	< 0.001
Region of residence			
Barisal	3.68	2.68–5.04	< 0.001
Chittagong	2.44	1.72–3.45	< 0.001
Dhaka	1.00		
Khulna	2.47	1.79–3.41	< 0.001
Mymensingh	1.58	1.15–2.17	< 0.01
Rajshahi	2.06	1.48–2.87	< 0.001
Rangpur	2.71	1.96–3.76	< 0.001
Sylhet	1.91	1.35–2.70	< 0.001
***Random effects terms: variance and 95% CI***			
Cluster level	0.72	0.60–0.86	

*CI* confidence interval

### Prevalence of diarrheal diseases in Bangladesh

Table 4 presents the results of the Poisson regression model of prevalence of diarrheal diseases among 8433 children in Bangladesh. The diarrheal disease prevalence was higher in unimproved sanitation facilities, which was 4.87 (4.30–5.52) and 4.49 (3.87–5.21) in improved sanitation facilities. The prevalence of diarrheal disease was 11.0% lower in SMS users than in unimproved sanitation users. After adjustment for confounding variables, the prevalence of diarrheal disease was almost the same (14.0%).

**Table 4:** Association between adequate sanitation and prevalence of diarrheal diseases among 8433 children in Bangladesh

Diarrheal diseases	Sanitation facilities	
	Unimproved	Improved
Percentage (95% CI)	4.87 (4.30–5.52)	4.49 (3.87–5.21)
Unadjusted PR (95% CI)	1.00	0.89 (0.73–1.09)
Adjusted PR (95% CI)^1^	1.00	0.86 (0.69–1.05)

*PR* prevalence ratio, *CI* confidence interval

^1^Adjusted confounding variables: age, gender, educational qualification, number of households, household socioeconomic status, place of residence, region of residence

## Discussion

Our study findings demonstrated that indicators with similar levels of overall coverage often have very different degrees of inequality. Additionally, older household heads, higher education, and richest socioeconomic status were potential factors for having better improved sanitation facilities. Socio-economic inequalities are greater in rural areas than in urban areas in Bangladesh. Concerning urban/rural disparities, access to improved sanitation is much higher in urban areas than in rural areas. The prevalence of diarrhea is lower in SMS users than in unimproved sanitation users.

Among the eight administrative divisions, the lowest sanitation coverage was observed in Mymensingh (32.9%), which is not unlikely as this division experiences river floods annually [24]. Water supply and sanitation conditions are seriously affected during floods, as they include multiple water-borne diseases [25]. This division also has the highest poverty level in Bangladesh [26], which is believed to have a strong impact on sanitation coverage. Poor people, particularly slum dwellers, suffer from inadequate basic infrastructure facilities, such as unimproved and unhygienic sanitation [27]. A substantial number of households have to share a few pit latrines in most slum areas [27]. Typically, approximately one-third of slum households use open holes [27].

In the present study, older household heads, higher education, and richest socioeconomic status were associated with SMS. These findings are consistent with those of previous studies in Bangladesh and other low-income countries [28]. Older and higher-educated household heads have better sanitation access, which could be because wisdom increases with age and experience [29] and education can foster wisdom [30]. They may also be attributed to the greater consciousness of their own health and hygiene. Richest households were found to have better access to improved sanitation facilities as rich people are more likely to afford them financially, and this supports the statement from another study that socioeconomic factors affect access to sanitation in LMICs, especially in rural areas [31].

In this study, the prevalence of diarrhea was 14.0% lower in SMS users than in unimproved sanitation users. This finding is consistent with that of a previous reports and a systematic review and meta-analysis [32, 33]. Wolf et al. reported that sanitation intervention reduced the prevalence of diarrhea by 25.0%, with evidence for greater reductions when high sanitation coverage was reached [33]. Waddington et al. reported that sanitation interventions reduced the prevalence of diarrhea by 37.0% [32]. Contrarily, the present finding is not consistent with a previous study from Bangladesh, which found that combined access to improved water and sanitation reduced the incidence of diarrhea; however, improved sanitation alone had no statistically significant impact on the incidence of childhood diarrhea [34]. However, the previous study was based on the BDHS conducted in 2007, including 8585 children [34]. The present study was based on 2017–2018 BDHS data and sanitation facilities, and diarrheal diseases may have substantially changed over the last several years. The present study with a large sample size suggests that improved sanitation facilities can lower the prevalence of diarrhea.

## Strengths and limitations

The findings of this study should be interpreted in light of some limitations. One limitation is the potential for recall bias as the data are self-reported. The information of sanitation and diarrhoea are self-reported or mother reported, which could be prone to recall bias. However, the present findings are in the same direction of previous estimates and thus could not be ruled out as a possible explanation for the findings of this study. Another limitation is that the DHS was conducted in Bangladesh a few years ago. Equality in access to improved sanitation facilities and diarrheal diseases has substantially changed over the last few years [35]. However, our study provides the basis to strengthen the worldwide evidence on the importance of equality of coverage of sanitation facilities for the lives of all people, with specific attention to the poor. Sanitation systems are strongly linked to the overall health status of the people of Bangladesh, and they could reduce deaths and disease burden associated with sanitation and hygiene risk factors.

## Conclusion

In conclusion, the present study found substantial inequalities in access to improved sanitation at both national and sub-national levels of Bangladesh, with the prevalence of diarrhea being lower among SMS users. These findings may help policy makers to prioritize adequate resource allocation for reducing inequality and expanding the coverage of improved sanitation in Bangladesh.

## Data Availability

Data are available from a public open-access repository. The relevant data were obtained from the MEASURE DHS and are available from the Demographic Health Surveys Program at https://dhsprogram.com/data/available-datasets.cfm. The title of the dataset is the Bangladesh Demographic and Health Survey (Bangladesh 2017–2018).

## Abbreviations

BDHS: Bangladesh Demographic and Health Survey
CI: Confidence interval
OR: Odds ratio
PSU: Primary sampling unit
RII: Relative index of inequality
SII: Slope index of inequality
SDG: Sustainable development goal
WASH: Water, sanitation, and hygiene
